# Mesenchymal stromal cells secretory pattern contributes to oncoinflammatory bone marrow microenvironment in polycythemia vera

**DOI:** 10.1016/j.htct.2025.106224

**Published:** 2025-11-29

**Authors:** Juçara Gastaldi Cominal, Maira da Costa Cacemiro, Giovana Michelassi Berbel, Rifkath Marie Laurance Rahimy, Gisele Vieira Rocha, Dalila Lucíola Zanette, Maria Carolina Oliveira, Lorena Lobo de Figueiredo-Pontes, Kelen Cristina Ribeiro Malmegrim, Fabíola Attié de Castro

**Affiliations:** a Department of Clinical Analyses, Toxicology and Food Science, School of Pharmaceutical Sciences of Ribeirão Preto, University of São Paulo, Ribeirão Preto, SP, Brazil; b Instituto Carlos Chagas /Fiocruz PR, Curitiba, PR, Brazil; c Gonçalo Moniz Institute, Oswaldo Cruz Foundation (IGM-FIOCRUZ/BA), Salvador, Bahia, 40296-710, Brazil; d D’Or Institute for Research and Education (IDOR), São Rafael Hospital Center for Biotechnology and Cell Therapy, Salvador, 41253-190, Brazil; e Center for Cell-based Therapy, Regional Blood Center of Ribeirão Preto Medical School, University of São Paulo, Ribeirão Preto, SP, Brazil; f Division of Rheumatology, Allergy and Immunotherapy, Department of Internal Medicine, Ribeirão Preto Medical School, University of São Paulo, Ribeirão Preto, Brazil; g Division of Hematology, Hemotherapy and Cellular Therapy, Department of Medical Imaging, Hematology, and Clinical Oncology, Ribeirão Preto Medical School, University of São Paulo, Ribeirão Preto, Brazil

**Keywords:** Polycythemia vera, Hematopoietic niche, Mesenchymal stromal cells, Neoangiogenesis, Immune modulation

## Abstract

**Introduction:**

Polycythemia vera is a myeloproliferative neoplasm marked by an increased proliferation of erythroid mature and precursors cells in bone marrow and peripheral blood. The pathophysiology is linked to the presence of the JAK2 driver mutation, epigenetic deregulation, and alterations in the bone marrow hematopoietic niche. Multipotent mesenchymal stromal cells (MSC) in the bone marrow, which are crucial for maintenance and development of hematopoietic stem cells, play a role in the communication between neoplastic cells and resident bone marrow cells by releasing various mediators that either suppress or promote tumor progression. These mediators include several essential immunomodulatory molecules, pro-angiogenic and growth factors. We hypothesized that MSC from polycythemia vera patients (Patient Group) would exhibit distinct properties compared to those from healthy donors (Control Group), thereby influencing the hematopoietic niche and contributing to disease pathogenesis.

**Methods:**

This study characterized MSC from patients, focusing on their secretory, proteomic, and phenotypic properties.

**Results:**

MSC from the Patient and Control Groups had similar immunophenotypes and multipotentiality. However, MSC from the Patient Group exhibited reduced immunomodulatory properties, and released distinct soluble immune and angiogenic mediators when compared with the Control Group. Global proteomic analysis revealed that MSC from patients presented upregulated expressions of *FAM175B, VP526A, CTTN, MAP4, BAX*, and *TPD52L2* but a downregulated *TNC* expression. These results indicate that MSC contribute to the inflammation pattern in the hematopoietic niche. The secretory and proteomic profile of MSC from patients, indicate that these cells may influence immune cell function, induce neoangiogenesis, and alter cell-to-cell interactions within the bone marrow, thereby fostering a pro-tumor microenvironment and favoring disease pathogenesis.

**Conclusion:**

These findings highlight the potential of targeting MSC-mediated pathways as a therapeutic strategy in polycythemia vera.

## Introduction

Multipotent mesenchymal stromal cells (MSCs) are key elements that regulate hematopoietic stem cells (HSC) and resident BM cells in the hematopoietic niche [1,2]. They are known for their self-renewal ability and potential to differentiate into bone, cartilage, and fat cells [1,2]. The paracrine effect of MSCs is related to the production of stem cell factor (SCF), granulocyte-macrophage colony-stimulating factor (GM-CSF), vascular endothelial growth factor (VEGF), platelet-derived growth factor (PDGF), indoleamine 2,3-dioxygenase (IDO), prostaglandin E2 (PGE2), C-X-C motif chemokine ligand 12 (CXCL12), and interleukin (IL)-1, −6, −7, −8, −11 and −15 [3, 4, 5, 6, 7]. These mediators contribute to cell proliferation and differentiation, immunomodulation and angiogenesis 6, 7.

MSCs play a role in the communication between neoplastic and resident BM cells by engaging multiple signaling pathways and release various mediators that can either suppress or promote tumor progression [8]. In myelodysplastic syndromes, MSCs favor the inflammatory milieu, immune system suppression, and disease progression [9]. Mice lymphoma-derived MSCs release CCL2, which increases the proliferation of tumor cells and attracts immunosuppressive cells to the lymphoid tissues [10].

On the other hand, MSC antitumor action is associated with angiogenesis inhibition through the activation of endothelial cell apoptosis in melanoma [11]. In a chronic myeloid leukemia model, MSCs attenuate K562 proliferation by inhibiting Wnt signaling [12]. In myeloproliferative neoplasms (MPN) the TGF-β1 produced by monocytes or macrophages convert MSCs into contractile α-SMA+ myofibroblasts that contribute to BM fibrosis [13,14]. In particular, BM alterations in polycythemia vera (PV) patients are marked by an inflammatory profile with elevated levels of inflammatory cytokines, chemokines and pro-angiogenic factors when compared with BM from apparently healthy donors (HD) [15]. These alterations potentially interfere with hematopoiesis, neoangiogenesis, cell-to-cell interactions, and cell-stroma interactions in the BM from MPN patients that contribute to a pro-tumor microenvironment and disease pathogenesis [15, 16, 17, 18].

In this context, our hypothesis is that there is an imbalance between MSC and neoplastic cells in the bone marrow hematopoietic niche in PV. Thus, this study examined the immunophenotype, proteomic and secretory profile of bone marrow MSCs from PV patients to describe their potential contribution to the inflammatory niche in this MPN.

## Material and methods

### Subjects

Samples were collected from apparently healthy BM donors and MPN patients at the Ribeirão Preto Medical School Hospital, University of São Paulo (HC-FMRP/USP). The study included five HD volunteers and seven PV patients, with the latter diagnosed according to the 2016 WHO criteria [19]. All PV patients were JAK2V617F-positive, newly diagnosed, and therefore not under any treatment or using antiplatelet drugs at the time of bone marrow collection. Moreover, their medical records did not report any major comorbidity, such as diabetes mellitus or hypertension (Table 1).

**Table 1:** Clinical characteristics of patients with polycythemia vera (PV) and healthy donors (HD).

	**PV** (*n* = 7)	**HD** (*n* = 5)
Age - years (range)	61 (31–66)	29.6 (24–37)
Gender - n (male %)	4 (57.14)	3 (60.0)
Risk - n (%)		
High	5 (71.43)	
Low	2 (28.57)	
Vascular events - n (%)		
Yes	3 (42.86)	
No	4 (57.14)	
Bone marrow fibrosis rate - n (%)		
0	3 (42.86)	
1	4 (57.14)	

The study was approved by the Ethics Committee for Human Research of the School of Pharmaceutical Sciences of Ribeirão Preto (protocol CAAE 55,545,716.6.0000.5403).

### Bone marrow mesenchymal stromal cell isolation and culture

Bone marrow mononuclear cells (BMMC) were separated by gradient centrifugation using Ficoll® Paque Plus (Sigma-Aldrich), according to the manufacturer’s instructions. BMMC were collected from the upper layer, washed twice with phosphate buffered saline (PBS), and centrifuged at 300 x *g* for 10 min at room temperature. The cells from the pellet were counted and then seeded at a density of 2–4 × 10^7^ cells in T75 cm² flasks. MSCs adhered to the plastic surface and grew, while the other cell types were eliminated during medium changes and cell passages.

BM-MSCs were maintained in base medium alpha-mem (GIBCO™, Thermo Fisher Scientific) supplemented with 15 % fetal bovine serum (HyClone, Cytiva), 100 U/mL penicillin, 100 µg/mL streptomycin and 2 mM L-glutamine (GIBCO™, Thermo Fisher Scientific), and cultured under 5 % CO_2_ at 37 °C. For the assays, MSCs were used at passages 3 and 4.

### Bone marrow mesenchymal stromal cell immunophenotyping

BM-MSC immunophenotyping was performed by flow cytometry (FACSCalibur™, BD Biosciences) using antibodies against: CD13 (clone WN15), CD29 (clone MAR4), CD31 (clone WM59), CD34 (Clone 8G12), CD44 (clone M1), CD45/14 (clone 2D1/MɸP9), CD49e (clone IIA1) CD54 (clone HA58), CD73 (clone AD2), CD90 (clone 5E10), CD105 (clone 266), CD166 (clone 3A6), and HLA-DR (clone G46-6) (BD Biosciences).

### In vitro adipogenic differentiation assay

BM-MSCs (6 × 10^3^) were cultured in 24-well plates under adipogenic conditions using StemPro™ Adipogenesis differentiation kit (GIBCO™, Thermo Fisher Scientific). Then, cells were washed twice with PBS and fixed in 4 % paraformaldehyde solution for 30 min. Cells were rinsed three times with distilled water, and once with 70 % (v/v) ethanol. Then, cells were stained with Sudan III (Merck, Sigma-Aldrich) for 30 min. Subsequently, cells were rinsed once in 70 % (v/v) ethanol, twice in distilled water, and then counterstained with Hematoxylin-Mayer solution (Merck, Sigma-Aldrich) for 3 min. After the Hematoxylin-Mayer staining, the cells were gently washed three times in distilled water and then kept in fresh water until they turned blue (from one to three minutes). Images were captured with an CX30 optical microscope (Olympus) at 10x magnification.

### In vitro osteogenic differentiation assay

BM-MSCs (6 × 10^3^) were cultured in 24-well plates for 14 days under osteogenic conditions with StemPro™ Osteogenesis Differentiation kit (GIBCO™, Thermo Fisher Scientific). Then, cells were washed twice with PBS and fixed in 4 % paraformaldehyde solution for 30 min. Cells were rinsed three times with distilled water and incubated in 5 % (w/v) silver nitrate solution (Merck, Sigma-Aldrich) for 60 min while exposed to a 100 W incandescent light. The silver nitrate solution was removed and cells were rinsed three times with distilled water. Cells were incubated with 5 % (w/v) sodium thiosulfate solution (Merck, Sigma-Aldrich) for 2 min and washed three times with distilled water. Then, cells were counterstained with hematoxylin-Mayer solution (Merck, Sigma-Aldrich) for 3 min and gently washed three times with water. Images were captured with an optical microscope CX30 (Olympus) at 10x magnification.

### Isolation of peripheral blood mononuclear cells

Peripheral blood mononuclear cells, used in co-cultivation and in inhibition of T-lymphocyte proliferation assays, were isolated from peripheral blood from two HD, using gradient centrifugation with Ficoll® Paque Plus (Sigma-Aldrich).

### Inhibition of T-lymphocyte proliferation

CD4 and CD8 T-lymphocytes, isolated from peripheral blood of two HD, were co-cultured with BM-MSC at different ratios to analyze inhibition of cell proliferation. Peripheral blood mononuclear cells **(**PBMC) were suspended in 4 mL of 0.1 % (w/v) bovine serum albumin (MERCK, Sigma-Aldrich) solution in PBS and stained with 5 µM of 5-(6)-Carboxyfluorescein diacetate N-succinimidyl ester (CFSE; MERCK, Sigma-Aldrich) for 10 min at 37 °C. Next, PBMC labeled with CFSE (PBMC^CFSE^) were centrifuged at 400 x *g* for 10 min at 4 °C, and washed twice with 20 mL of complete Roswell Park Memorial Institute Medium 1640 followed by centrifugation at 400 x *g* for 10 min at 4 °C.

BM-MSCs were seeded in a 48-well culture plate at five concentrations: 1.25 × 10^5^ cells/mL (ratio 1:2), 5 × 10^4^ cells/mL (ratio 1:5), 2.5 × 10^4^ cells/mL (ratio 1:10), 1.25 × 10^4^ cells/mL (ratio 1:20) and 0.5 × 10^4^ cells/mL (ratio 1:50). Cells were cultured in 10 % fetal bovine serum α-MEM media under 5 % CO_2_ for 8 h at 37 °C. The supernatant was removed, and 1 mL of PBMC^CFSE^ was seeded per well (2.5 × 10^5^ cell/mL). To stimulate PBMC^CFSE^ cell proliferation, 3.12 µL of CD3/CD28 magnetic beads (Dynabeads Human T-Activator CD3/CD28, Thermo Fisher Scientific) were added to the mixture at the 1:1 bead:T-cell ratio recommended by the manufacturer. The beads are necessary to activate CD4 and CD8 proliferation.

The plate was incubated under 5 % CO_2_ for five days at 37 °C. The supernatant was removed and centrifuged at 400 x *g* for 10 min at 4 °C. The resulting supernatant was aliquoted and stored at −80 °C to further analyze soluble mediators. The cell pellet (PBMC^CFSE^) was suspended in PBS, stained with anti-CD4 and anti-CD8 antibodies (BD Biosciences), and analyzed in a FACSCalibur™ flow cytometer (BD Biosciences). PBMC^CFSE^ not stimulated with beads were used as the negative control. The percentage of CD4 and CD8 cell proliferation inhibition was calculated by the proliferation ratios of CD4:CD8 cells cultured alone or in combination with different MSC concentrations.

### Bone marrow mesenchymal stromal cell doubling time

BM-MSCs at passages 3 and 4 were seeded in a 24-well plate, at a density of 3000 cells/cm² with one mL of complete base medium, and cultured at 37 °C for seven days under 5 % CO_2_. Three wells were trypsinized with 0.25 % trypsin solution (GIBCO™, Thermo Fisher Scientific) every 24 h. The BM-MSC concentration and viability were determined using 0.4 % trypan blue (GIBCO™, Thermo Fisher Scientific).

The cell doubling time was calculated using the following equation: DT = (*t* x log2) / (log(*n*/*n_0_*)), where DT = doubling time, *t* = cultivation time (days), *n* = total cells at the end of seven days, and *n_o_* = total cells in the beginning of the experiment (Day 0). The exponential cell growth curve was plotted using the GraphPad Prism 6.0 (Dotmatics) software.

### Quantification of soluble immune and angiogenic mediators

Supernatants from co-cultures of BM-MSCs and PBMCs at 1:10 ratio were used to quantify epidermal growth factor (EGF), fibroblast growth factor 2 (FGF-2), Fms-related tyrosine kinase 3 ligand (Flt-3 L), interferon alpha (IFN-α), macrophage-derived chemokine (MDC), interleukin 1 beta (IL-1β), interleukin 6 (IL-6), tumor necrosis factor-alpha (TNF-α), and vascular endothelial growth factor (VEGF) using the multiplex human customized Magnetic Luminex® Assay (R&D Systems), according to the manufacturer’s instructions. The samples were analyzed in a MX® Luminex (MERCK) and the concentration was determined using xPONENT (Luminex, MERCK, Sigma-Aldrich).

### Proteomic analysis of bone marrow mesenchymal stromal cells from polycythemia vera patients and healthy donors

Protein was extracted from cultured MSCs at passage 3. Pelleted cells were washed twice with PBS to remove fetal bovine serum and then suspended in cell lysis buffer with four protease inhibitors. After centrifugation, the supernatant was used to quantify proteins by a fluorescence-based method using the Qubit Protein Assay kit and the Qubit fluorimeter (Thermo Fisher Scientific). Ten micrograms of protein lysates were applied in denaturant 10 % polyacrylamide gel (SDS-PAGE) and stained with Coomassie. The gel bands were cut, discolored, dehydrated, and dried. The dry fragments were reduced and alkylated, and the resulting liquid part was discarded. Then the fragments were digested, dehydrated, dried (twice), further incubated with a trypsin solution, washed, and digested. The liquid part was mixed with the extraction solution (water, 3 % TFA, 30 % acetonitrile) and incubated. The supernatants were transferred to new tubes and incubated twice with acetonitrile, followed by drying using a SpeedVac until the volume was reduced to 10–20 % of the original. The peptides were purified with StageTips-C18, dried and diluted in aqueous/dilution solution for analysis in a LC-MS/MS equipment (Liquid Chromatograph Ultimate 3000, Thermo Scientific). The parameters used were gradient phase A: 0.1 % formic acid, 5 % DMSO and phase B: 0.1 % formic acid, 5 % DMSO in acetonitrile, under the flow rate of 250 nL/min, with linear gradients of 5–40 % of phase B in 120 min. The analytical columns were 15 cm length with internal diameter of 75 µm. Mass spectrometry was performed in a hybrid LTQ Orbitrap XL ETD, Thermo Scientific Full Scan (MS1) with full scan window (*m/z*) of 300.0–2000.0, resolution of 60,000, lock mass of 401.922718 *m/z*.

Perseus software was used to analyze proteomic data according to the developer's instructions. The fluorescence intensity recorded in the mass spectrometer was normalized, values that were not detected in any sample of a group were eliminated, and groups of two samples at a time were compared using t-test [20].

### Gene ontology biological process analysis

Gene Ontology analysis was performed using the Webgestalt online tool (https://www.webgestalt.org/) with the following settings: Over-representation Analysis (ORA) method, Gene Ontology Database non-redundant Database, with significance level cut-off false discovery rate (FDR) <0.05, and viewint the top ten categories. The 102 differentially expressed proteins (DEPs) were loaded in separate runs to compare DEPs of BM-MSCs from PV patients to BM-MSC from HD: upregulated DEPs (58 proteins) and downregulated DEPs (44 proteins) [21].

### Statistical analysis

Proteomic data were analyzed and graphs plotted using GraphPad Prism version 6.01 and Excel for Microsoft 365. The Kruskal-Wallis test with Dunn's post-test was used for nonparametric analysis of variance to compare mean values across experimental conditions, and the Mann-Whitney test was used for two-group comparisons. Proteomics statistics were based on label-free quantification (LFQ) of protein abundance. Raw mass spectrometry data were analyzed using MaxQuant software, and proteins with FDR ≥1 % were filtered out. The resulting "proteinGroups.txt" table was imported into R (version 4.2) for differential protein expression analysis using the Differential Enrichment Analysis of Proteomics data package. Contaminant and reverse proteins were removed, and data were filtered for proteins with LFQ >0 in at least one group. The LFQ intensities were normalized and imputed with random Gaussian distribution draws around a minimal value (p-value <0.01). Differential enrichment analysis was performed using the Limma function in the Differential Enrichment Analysis of Proteomics data package, selecting proteins with p-adjust ≤0.05 and log2 fold change >1 [22,23].

## Results

### Bone marrow mesenchymal stromal cell characterization

**Figure 1:**
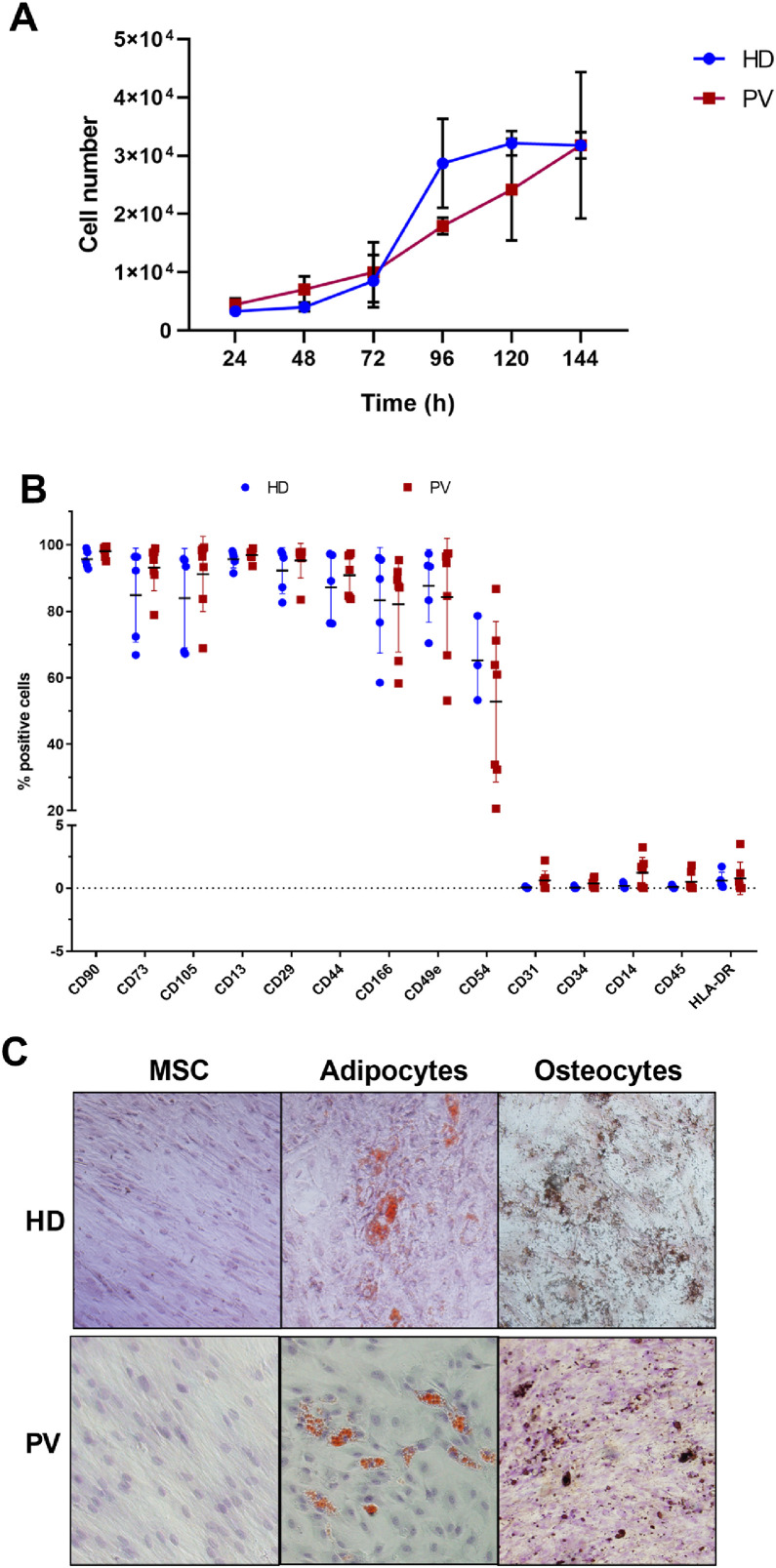
Characterization of bone marrow mesenchymal stromal cells (BM-MSC) of polycythemia vera (PV) patients and healthy donors (HD). **A.** BM-MSC growth curve, where data were expressed as means (PV: *n* = 4; HD: *n* = 3). **B.** BM-MSC immunophenotype, expressed as percentage of positive cells relative to 10,000 events. The number of the samples varied according to the marker **C.** Representative images of multipotential analysis. Left: BM-MSC grown in base medium (α-MEM supplemented with 10 % fetal bovine serum) and stained with hematoxylin. Center: MSC after 12 days of growth in adipogenic medium. Lipid accumulation in adipocytes (orange) stained with Sudam III and counterstained with hematoxylin. Right: MSC after 15 days of growth in osteogenic medium. Calcium deposits in osteocytes (brown) stained with von Kossa stain and counterstained with hematoxylin.

The proliferative potential of BM-MSCs were examined from five PV patients and three HD. The BM-MSC doubling time was 46.2 and 55.5 h in HD and PV patients, respectively (Figure 1A) and the growth rate (μ) was 0.36 and 0.30/day in BM-MSC from HD and PV patients, respectively.

The immunophenotypic analysis revealed that BM-MSC from both PV patients and HD highly expressed the cell surface markers CD90, CD73, CD105, CD13, CD29, CD44, CD166 and CD49e, intermediately expressed CD54, and weakly expressed HLA-DR, CD31, CD34, CD14, and CD45 (Figure 1B).

There were no differences between the BM-MSC immunophenotypes from PV patients and HD (Figure 1B), and the BM-MSC from both groups were able to transdifferentiate into osteogenic and adipogenic cell lines (Figure 1C).

### Bone marrow mesenchymal stromal cells from polycythemia vera patients alter CD8^+^*T* lymphocyte proliferation

**Figure 2:**
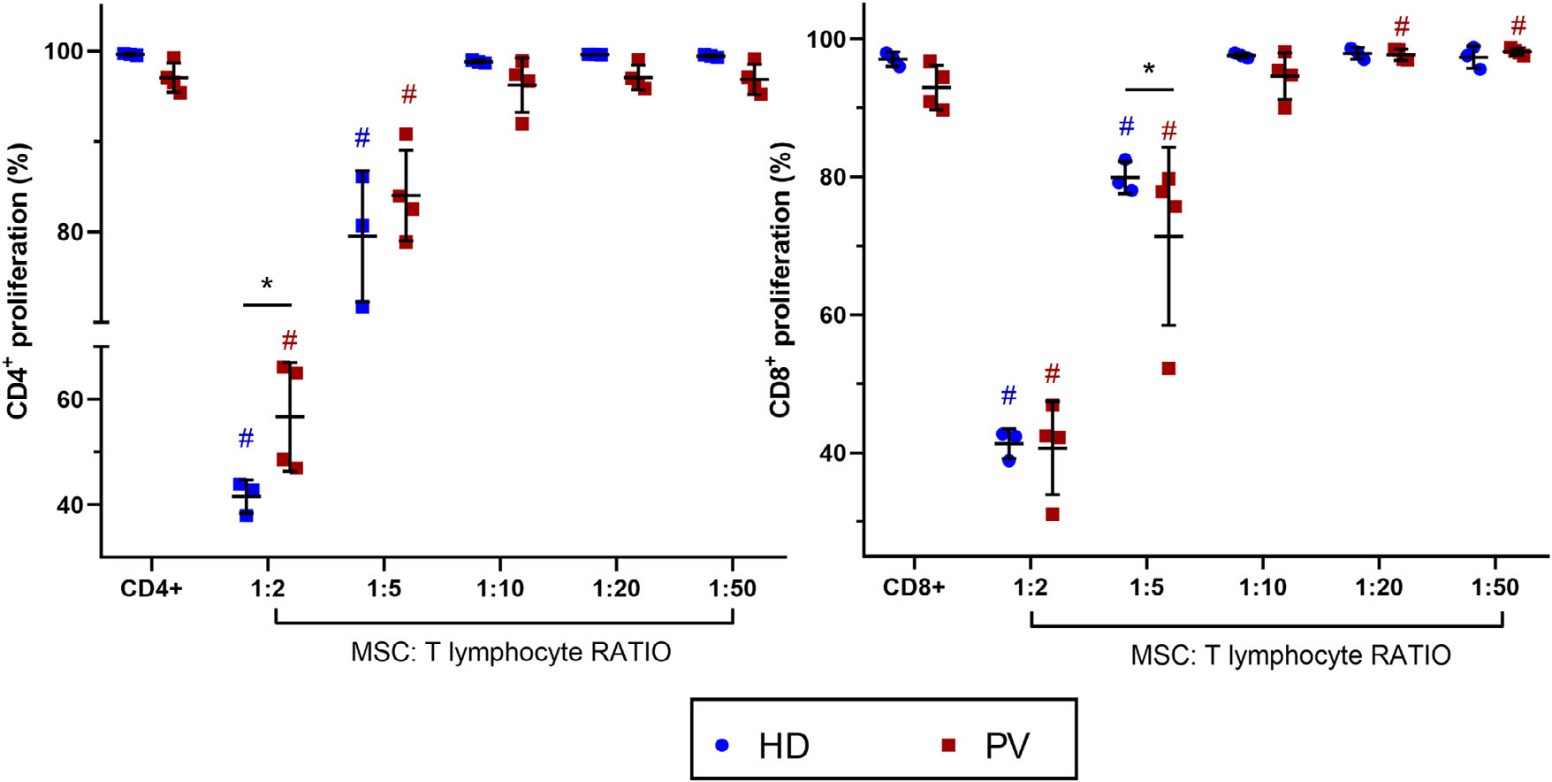
Bone marrow mesenchymal stromal cells (BM-MSC) immunomodulation of CD4^+^ and CD8^+^ proliferation. Comparison between CD4^+^ and CD8^+^ proliferation cultured with and without BM-MSC from polycythemia vera patients (PV: *n* = 4) and healthy donors (HD: *n* = 3). T lymphocytes from a blood donor were used. P-value ≤0.05 indicated statistical significance (multiple linear regression analysis). All the experimental conditions were performed in triplicate. Dark blue # means significant differences between HD with CD4^+^ or CD8^+^ alone. Red # means significant differences between PV patients with CD4^+^ or CD8^+^ alone. Black * means significant differences between PV and HD.

BM-MSC were co-cultured with activated CD4^+^ and CD8^+^ *T* lymphocytes to evaluate their immunomodulatory effects on T cell proliferation (Figure 2). In both PV patients and HD groups, T cell proliferation (CD4^+^ and CD8^+^) decreased at high BM-MSC:lymphocyte ratios (1:2 and 1:5). Notably, BM-MSC from PV patients modulated CD8+ *T* cell proliferation in a concentration-dependent manner. At high BM-MSC:lymphocyte ratios (1:2 and 1:5), CD8^+^ *T* cell proliferation was reduced, while at low ratios (1:20 and 1:50), it was enhanced. At a BM-MSC:lymphocyte ratio of 1:10, CD8^+^ *T* cell proliferation was similar to that of CD8^+^ *T* cells cultured alone, indicating a balance and lack of immunomodulation. When comparing suppression effects between groups, BM-MSC from HD more effectively inhibited CD4^+^ *T* cell proliferation at a 1:2 BM-MSC:lymphocyte ratio. Conversely, BM-MSC from PV patients more effectively decreased CD8^+^ *T* cell proliferation at a lower BM-MSC:lymphocyte ratio (1:5) compared to BM-MSC from HD. At lower BM-MSC:lymphocyte ratios, there were no significant differences in the immunomodulatory effects on CD8^+^ and CD4^+^ *T* cells between HD and PV.

### Bone marrow mesenchymal stromal cell secretory profile

**Figure 3:**
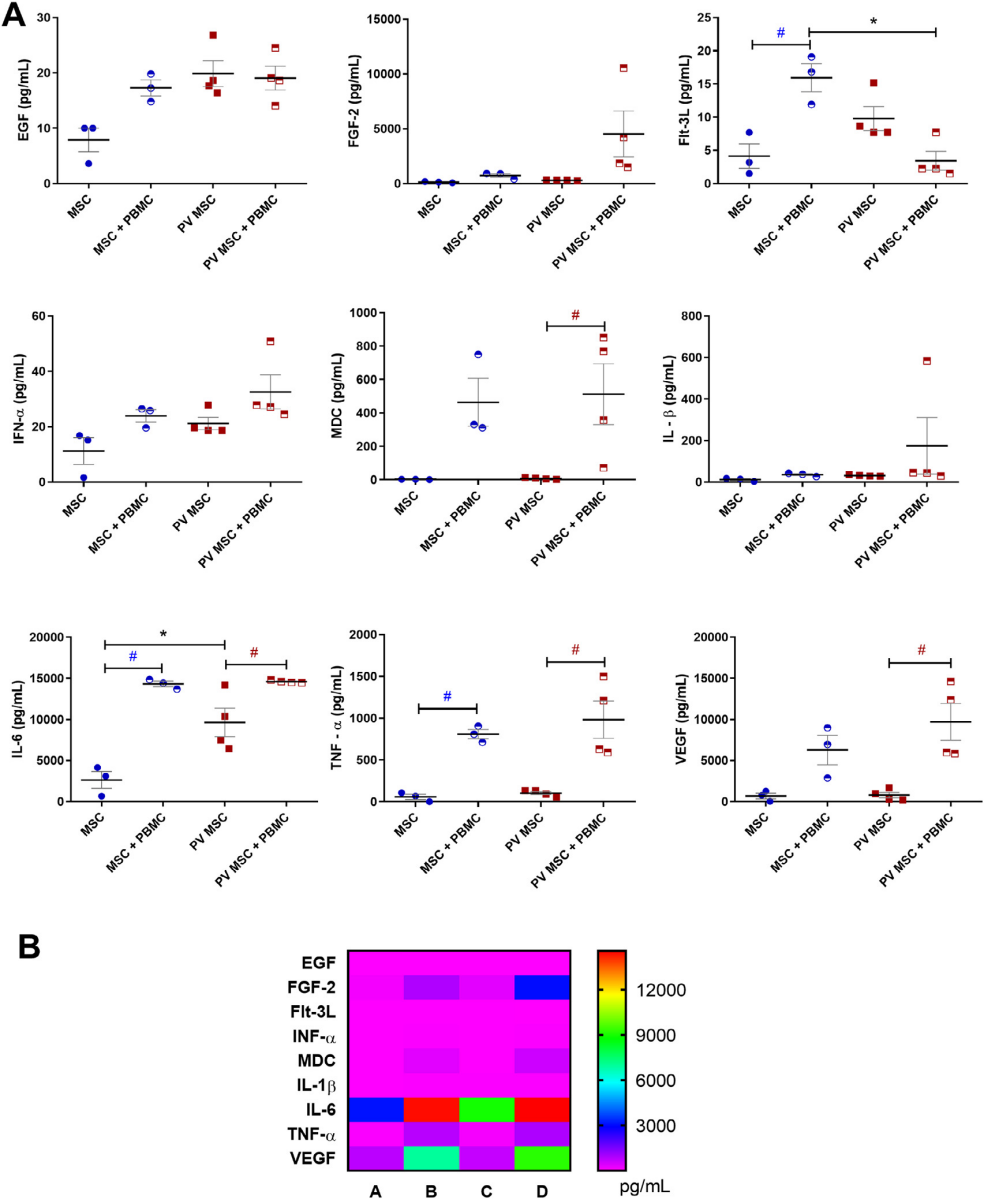
Quantification of cytokines and angiogenic factors in bone marrow (BM) multipotent mesenchymal stromal cells (MSC) and PBMC (peripheral blood mononuclear cells) under mono- and co-culture conditions. BM-MSC from patients with polycythemia vera (PV: *n* = 4) and from healthy donors (HD: *n* = 3) were cultured for 24 h in the presence or absence of PBMC activated with CD3/CD28 magnetic beads. **A:** Black * means significant differences between PV and HD MSC. Dark blue # means significant difference between MSC (HD) conditions. Red # means significant differences between PV MSC conditions. p-value <0.05 indicates statistical significance; * or # indicates p-value ≤0.05 (ANOVA followed by the Tukey test). **B:** Heatmap of cytokines and angiogenic factor concentrations (pg/mL) in (A) monoculture of BM-MSC from HD; (B) co-culture of BM-MSC from HD and PBMC; (C) monoculture of BM-MSC from PV patients; (D) co-culture of BM-MSC from PV patients and PBMC.

Inflammatory and angiogenic factors were quantified in the supernatants from mono- and co-cultures of BM-MSC and PBMC to examine the BM-MSC secretory profile (Figure 3). Compared with BM-MSC from HD, those from PV patients released higher concentrations of EGF (p-value ≤0.05) and IL-6 (p-value ≤0.01). Both BM-MSC groups – from HD (p-value ≤0.001) and PV patients (p-value ≤0.05) – released higher concentrations of IL-6 and TNF-α when they were co-cultured with PBMC.

BM-MSC from PV patients co-cultured with PBMC released (i) had higher macrophage-derived cytokine (MDC; p-value ≤0.05) and vascular endothelial growth factor (VEGF; p-value ≤0.01) levels, compared with BM-MSC from PV patients alone; and (ii) lower FMS-like tyrosine kinase 3 ligand (Flt-3 L; p-value ≤0.01) levels than MSC from HD co-cultured with PBMC (Figure 3A).

The categorical analysis of the angiogenic mediators produced by BM-MSC from HD revealed that they were altered and PBMC-dependent. No relevant levels of angiogenic factors were detected in the supernatant from monocultures of BM-MSC from HD. In contrast, the supernatant from BM-MSC from HD co-cultured with PBMC had high concentrations of EGF, FGF-2, Flt-3 L, IL-6, IL-1β, and VEGF (Figure 3B). The supernatants from BM-MSC from PV patients alone or co-cultured with PBMC presented high concentrations of angiogenic and immune cell mediators.

### Protein global expression in polycythemia vera mesenchymal stromal cells

**Figure 4:**
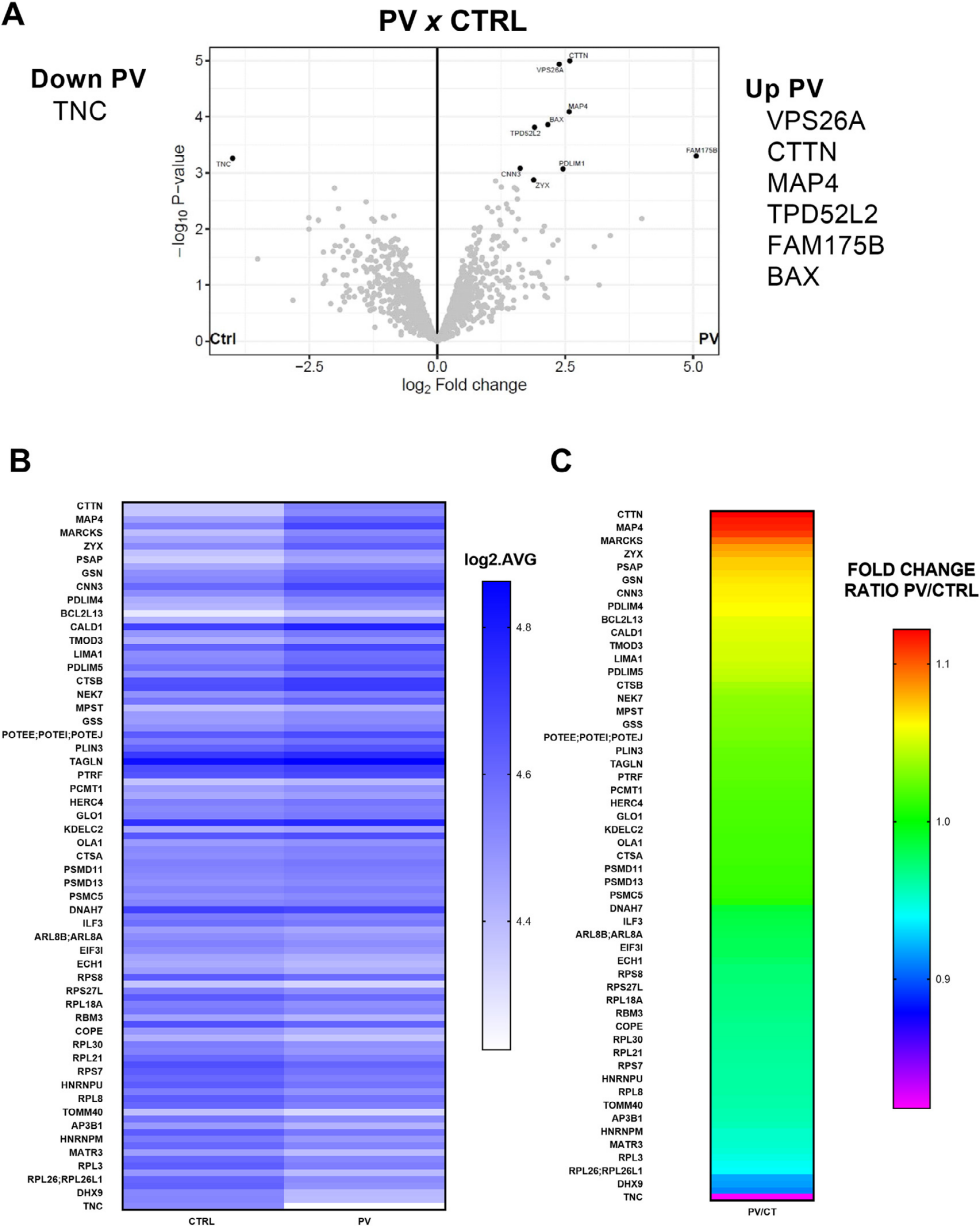
Proteomics analysis of bone marrow mesenchymal stromal cells (BM-MSC) from patients with polycythemia vera (PV: *n* = 4) compared with BM-MSC from healthy donors (HD: *n* = 3). **A:** Volcano Plots of proteins differentially expressed in MSC from PV patients compared to MSC from HD. R analysis (more stringent) was used to identify the differences. Six proteins were upregulated, and one was downregulated. **B:** Heatmap of Perseus analysis (less stringent). The number of differentially expressed proteins (p-value ≤0.05) in BM-MSC from PV patients was 102. Results expressed in log2 average. **C:** Heatmap of fold change ratio PV/HD from 102 differentially expressed proteins (Perseus analysis).

Proteomic analysis was carried out to compare BM-MSC from PV patients and HD using Perseus (less stringent) and R (more stringent) software systems (Figure 4). Compared with BM-MSC from HD (Controls, HD), the number of differentially expressed proteins (p-value ≤0.05) in BM-MSC from PV patients was 102 (Figure 4B-C). Just seven proteins were differentially expressed in BM-MSC from PV patients, when the R package was used for the most stringent analysis. The differentially expressed proteins in PV patients evidenced by the Volcano plots were upregulated FAM175B, VP526A, CTTN, MAP4, BAX, TPD52L2 and downregulated TNC (Figure 4A).

### Gene ontology biological process analysis

**Figure 5:**
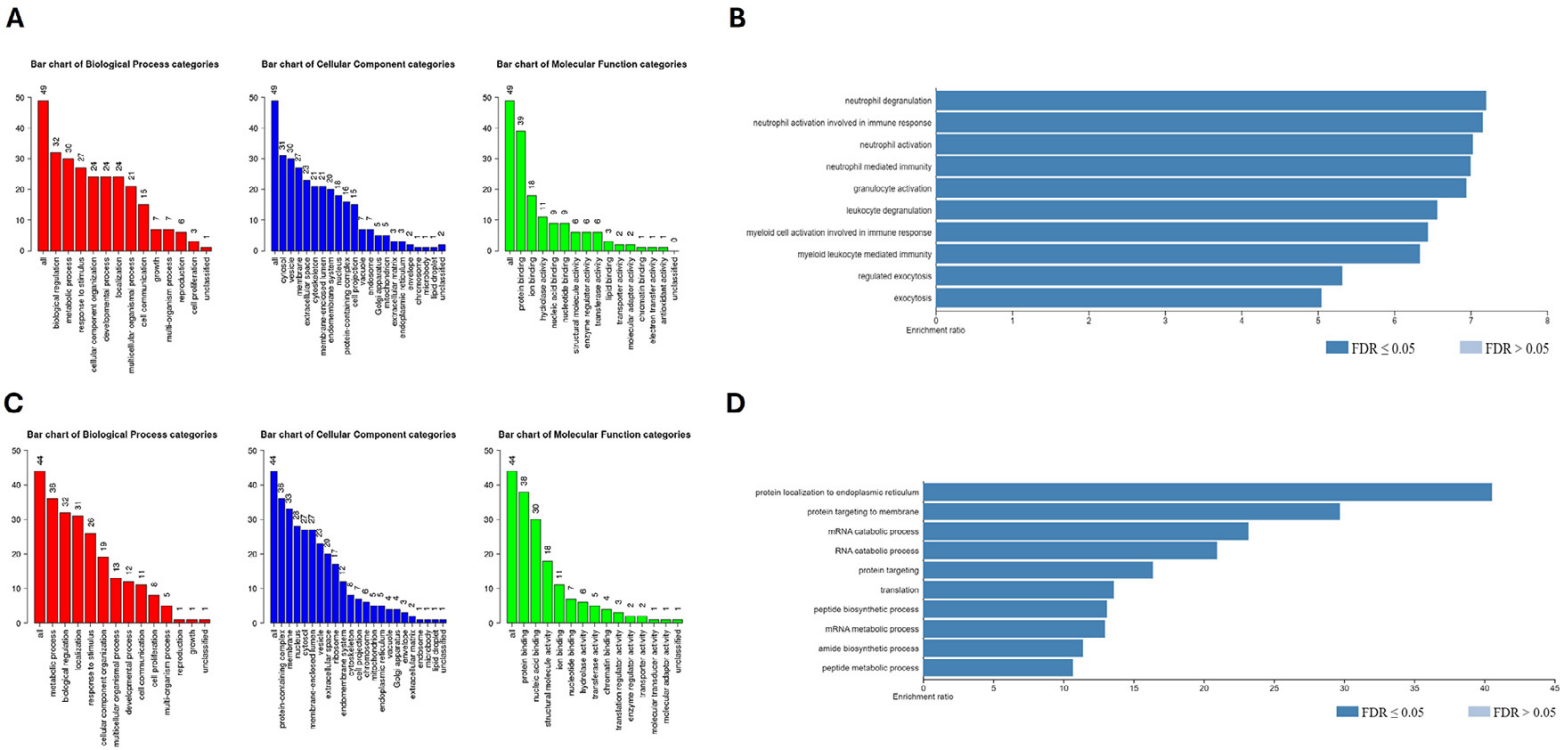
Gene ontology (GO) enrichment analysis of differentially expressed proteins (DEPs) in bone marrow mesenchymal stromal cells (BM-MSC) from polycythemia vera (PV) patients. **A:** GO categories enriched among upregulated proteins in PV-MSC, including biological processes, cellular components, and molecular functions. **B:** Functional enrichment of 58 upregulated DEPs, highlighting terms related to neutrophil-mediated activity. **C:** GO categories associated with 44 downregulated DEPs in PV-MSC, reflecting trends in reduced biological and molecular processes. **D:** Enrichment analysis of downregulated proteins showing pathways related to protein targeting the endoplasmic reticulum and mRNA/protein metabolism.

Figure 5A depicts the main gene ontology categories linked to the upregulated proteins in BM-MSC from PV patients. The three main biological processes detected in this group were biological regulation, metabolic processing, and response to stimulus. The enrichment analysis based on Biological Process and Gene Ontology Datasets of the 58 upregulated DEPs in BM-MSC from PV patients compared to BM-MSC from HD identified the top categories “neutrophil degranulation”, “neutrophil activation involved in immune response”, and “neutrophil activation” (Figure 5B). All the ten top categories were related to white blood cell activity and exocytosis. The 44 downregulated DEPs in BM-MSC from PV patients were subjected to the same analysis and the results for the biological processes were similar (Figure 5C). The enrichment analysis of the downregulated proteins in BM-MSC from PV patients identified the top category “protein targeting to endoplasmic reticulum”. All the top ten categories were involved in protein and mRNA metabolism (Figure 5D).

FDR: False discovery rate.

## Discussion

BM-MSC from the BM microenvironment not only regulate HSCs but also various resident cells associated with tumorigenesis and malignant transformation within the hematopoietic niche. They contribute to immune evasion through paracrine activity, promoting the growth and survival of neoplastic cells and activating angiogenesis [24, 25, 26].

BM-MSC from PV patients and HD exhibited similar morphology, immunophenotype, and multipotentiality. However, PV BM-MSC proliferated more slowly, with longer doubling times compared to those from HD. This is consistent with findings in MPN and other hematological diseases [27, 28, 29].

BM-MSC suppress the proliferation of immune cells, including dendritic cells, natural killer cells, and T lymphocytes, mainly through cell-to-cell contact and secretion of mediators like PGE2 and TGF-β [30,31]. In this study, BM-MSC from HD inhibited both CD4^+^ and CD8^+^ *T* cell proliferation in a concentration-dependent manner, with higher BM-MSC concentrations reducing proliferation and inhibiting mononuclear cell functions. PV BM-MSC showed similar effects but paradoxically enhanced T cell proliferation at lower concentrations, suggesting that they provide additional stimulation for T cell proliferation.

T cells are crucial for antitumor immunity, and their negative regulation is linked to immune evasion mechanisms favoring tumorigenesis [30, 31, 32, 33]. Data from this study show that BM-MSC exerted differential effects on immune cells depending on their concentration within the co-culture system. At high BM-MSC proportions, they inhibited immune cell functions, whereas low concentrations stimulated cell proliferation. This suggests that neoplastic cells may regulate MSC to release proliferative stimuli rather than suppress immune responses, thus aiding tumor progression.

The differential effects of PV BM-MSC on T cell proliferation highlight the role of the BM niche in immune modulation and tumor progression. Low BM-MSC ratios enhanced T cell proliferation, supporting a pro-tumor microenvironment and potentially contributing to MPN pathogenesis and oncoinflammation. Furthermore, cell-to-cell interactions in the BM reveal how MSC alter immune responses in PV and influence disease progression.

In PV patients, the BM microenvironment was marked by an inflammatory profile, with MSCs producing elevated levels of cytokines and chemokines, dysregulating hematopoiesis and favoring neoplastic cell proliferation [15]. The pro-inflammatory profile of MSCs (MSC1) has antitumor effects, while the immunosuppressive profile (MSC2) promotes tumor growth by inhibiting T cell activation 34, 35. The inflammatory microenvironment in PV patients likely biases MSC toward the immunosuppressive MSC2 profile, contributing to disease progression. Interestingly, angiogenic factors were undetectable in monocultures of BM-MSCs from HD, which may reflect their quiescent secretory profile under basal, non-inflammatory conditions. We hypothesize that these cells require interaction with immune cells or exposure to inflammatory signals, such as those present in PV, to activate their proangiogenic secretome. This reinforces the idea that the BM microenvironment plays a central role in modulating MSC behavior and should be taken into account when interpreting functional assays.

Proteomic analysis revealed that PV BM-MSC exhibited differential expression of proteins like CTTN, MAP4, TPD52L2, and BAX, associated with cell migration, intracellular transport, and apoptosis regulation. These proteins could play a role in MSC-mediated immune modulation and neoplastic cell evasion in PV.

The function of cortactin (CTTN), an actin-binding protein widely expressed in human cells, is associated with cell adhesion and migration. CTTN has recently been detected in different hematopoietic cells such as lymphocytes, dendritic cells, and macrophages. Its expression is upregulated in chronic lymphoid leukemia, acute lymphoid leukemia, and non-Hodgkin's lymphoma [36].

Upregulation of microtubule-associated protein 4 (MAP4) expression was also related to tumor invasion and migration, in addition to the worse prognosis of different solid tumors, such as lung adenocarcinoma [37] and breast cancer [38]. Its high expression correlates with tumor resistance to treatments with microtubule-targeting agents, such as vinca alkaloids, in lymphomas, breast cancer, and acute lymphoid leukemia 37, 38.

*TPD52L2* is the gene that encodes the protein known as tumor protein D54, which is a biomarker for breast tumors, different types of carcinomas, and lymphoid and acute myeloid leukemia [39]. This protein participates in the intracellular transport and membrane trafficking by intracellular nanoparticles 39, 40 that mediate integrin recycling and control cell migration and invasion [40].

Considering the abovementioned information the CTTN, MAP4, and TPD52L2 are essential for cell mobility, intracellular transport, and cell-to-cell interactions. Further research is required to unravel the precise mechanisms and functional implications of these proteins in MSCs.

The pro-apoptotic protein BAX was also upregulated in MSCs from PV patients. BAX is a pro-apoptotic member of the Bcl-2 family of proteins that regulate the activation of the apoptosis intrinsic pathway [41]. Recently, Pang et al. reported that low expressions of the apoptotic effectors BAK and BAX in MSCs impair cell death and reduce the immunosuppressive action of MSCs in a murine model of allergic asthma. In this model, MSC death is crucial for them to exert their functions [42]. In this sense, we speculate that high levels of BAX protein favor MSCs to suppress the proliferation and response of various immune cell subsets in the BM microenvironment and thereby contribute to PV neoplastic cell evasion from immune response.

BAX, a pro-apoptotic protein, was upregulated, which may help MSCs suppress immune responses in the BM microenvironment and support the evasion of PV cells from immune detection.

Gene Ontology analysis revealed that upregulated proteins in PV were enriched in processes linked to disease activity, particularly related to neutrophils, immunity, granulocytes, and exocytosis. Downregulated proteins were involved in transcription and protein translation, particularly ribosomal proteins and RNA splicing factors, which are often mutated in MPN [39].

Overall, these findings provide insights into the changes in the BM microenvironment in PV patients, improving our understanding of MPN pathophysiology and suggesting new therapeutic strategies targeting the inflammatory BM niche to restore immune balance and control disease progression. A potential limitation of this study is the age difference between PV patients and HD, as aging may modulate MSC functions. To address this issue, the data were reanalyzed including age as a covariate. This statistical reanalysis confirmed that age did not influence the outcomes of the study. These results support the robustness of our conclusions despite the age disparity between groups.

## Conflicts of interest

The authors declare no conflicts of interest.
